# Edema is not a reliable diagnostic sign to exclude small brain metastases

**DOI:** 10.1371/journal.pone.0177217

**Published:** 2017-05-11

**Authors:** Tanja Schneider, Jan Felix Kuhne, Paul Bittrich, Julian Schroeder, Tim Magnus, Malte Mohme, Malte Grosser, Gerhard Schoen, Jens Fiehler, Susanne Siemonsen

**Affiliations:** 1 Department of Diagnostic and Interventional Neuroradiology, University Medical Center Hamburg-Eppendorf, Hamburg, Germany; 2 Department of Neurology, University Medical Center Hamburg-Eppendorf, Hamburg, Germany; 3 Department of Neurosurgery, University Medical Center Hamburg-Eppendorf, Hamburg, Germany; 4 Department of Medical Biometry and Epidemiology, University Medical Center Hamburg-Eppendorf, Hamburg, Germany; University of South Alabama Mitchell Cancer Institute, UNITED STATES

## Abstract

No prior systematic study on the extent of vasogenic edema (VE) in patients with brain metastases (BM) exists. Here, we aim to determine 1) the general volumetric relationship between BM and VE, 2) a threshold diameter above which a BM shows VE, and 3) the influence of the primary tumor and location of the BM in order to improve diagnostic processes and understanding of edema formation. This single center, retrospective study includes 173 untreated patients with histologically proven BM. Semi-manual segmentation of 1416 BM on contrast-enhanced T1-weighted images and of 865 VE on fluid-attenuated inversion recovery/T2-weighted images was conducted. Statistical analyses were performed using a paired-samples t-test, linear regression/generalized mixed-effects model, and receiver-operating characteristic (ROC) curve controlling for the possible effect of non-uniformly distributed metastases among patients. For BM with non-confluent edema (n = 545), there was a statistically significant positive correlation between the volumes of the BM and the VE (P < 0.001). The optimal threshold for edema formation was a diameter of 9.4 mm for all BM. The primary tumors as interaction term in multivariate analysis had a significant influence on VE formation whereas location had not. Hence VE development is dependent on the volume of the underlying BM and the site of the primary neoplasm, but not from the location of the BM.

## Introduction

Brain metastases (BM) are the most common type of brain tumors in adults, accounting for 51% of cerebral neoplasms altogether [1]. BM are generally surrounded by vasogenic edema (VE) resulting from tumor-induced blood-brain barrier disruption and penetration of fluid with a high protein content into the white matter interstitial space [2, 3]. The presence of VE may significantly contribute to clinical worsening of patients with BM and may cause or exacerbate seizures, headaches, or focal neurologic deficits [4]. Magnetic resonance imaging (MRI) is the method of choice for imaging of BM and VE [5].

A number of textbooks state that degree of edema is unrelated to the volume of the underlying BM, but there is no prior systematic study examining the relationship between BM and concomitant VE volume [6–8]. This may be crucial for a better understanding of molecular mechanisms involved in edema formation and may also improve assessment of metastases sizes on unenhanced MRI in times of controversial discussions about Gadolinium deposition in brain tissue [9].

In this study, we aim to determine 1) the general volumetric relationship between BM and VE, 2) a threshold diameter above which a BM induces peritumoral edema, and 3) the influence of the primary tumor and location (supra-/infratentorial) of BM on VE development.

## Materials and methods

### Study cohort

**Fig 1:**
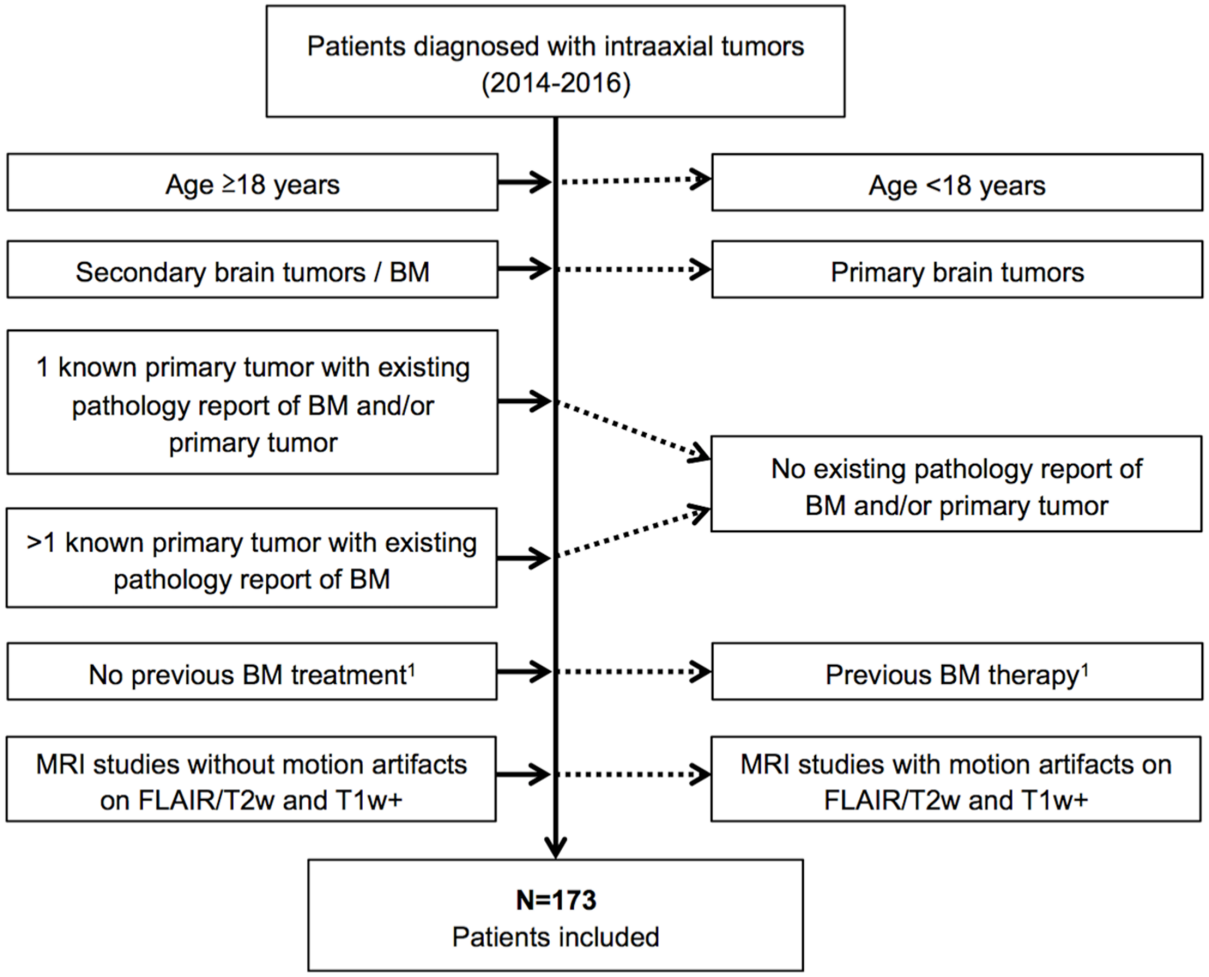
Flow-chart summarizing patient selection in the study. BM = brain metastases, MRI = magnetic resonance imaging.

This single center, retrospective study was conducted in compliance with the local ethics committee (Ethik-Kommission der Ärztekammer Hamburg, WF-018/15) with a waiver of informed consent. To collect cases, local MRI studies over a three-year period (01/2014-12/2016) were screened for the presence of secondary malignant intraaxial brain tumors. Inclusion and exclusion criteria are given in Fig 1. In total, 173 patients (90 males and 83 females) with 1416 BM were included.

In a second step, an electronic chart review of the hospital’s information system of eligible cases was performed. Metastases-related data including date of diagnosis (primary tumor and BM), type of primary neoplasm, patient age, and patient sex were further collected.

### MRI study protocol

MRI was performed using a 1.5 Tesla (Magnetom^®^ Sonata, Siemens Healthcare, Erlangen, Germany; Magnetom^®^ Symphony, Siemens Healthcare, Erlangen, Germany, and Magnetom^®^ Avanto, Siemens Healthcare, Erlangen, Germany) in 155 patients or a 3 Tesla scanner (Magnetom^®^ Skyra, Siemens Healthcare, Erlangen, Germany; Ingenia, Philips Medical Systems, Best, The Netherlands) in 18 patients. Imaging protocol always included non-contrast axial fluid-attenuated inversion recovery (FLAIR) or T2-weighted (T2w) turbo spin echo imaging. Following weight-adjusted Gadolinium injection, axial T1-weighted (T1w) spin echo with flow compensation and three-dimensional T1w gradient echo sequences were acquired. Sequence parameters (repetition time, echo time, inversion time, field of view, matrix, pixel size, slice thickness, interslice gap, and number of slices) varied among the different scanners.

### Image analysis

Image Analysis was performed with Analyze Software System 11.0 (Biomedical Imaging Resource, Mayo Clinic, Rochester, MN, USA) by a single reader (J. F. K.) [10]. For this purpose, non-confluent edemas (n = 545/865 edema positive BM) were contoured semi-manually on each FLAIR/T2w slice (total lesion region of interest (ROI), in ml), including the corresponding BM. Separately, all BM were segmented on contrast-enhanced T1w images (BM ROI, in ml). VE volume was then determined by subtracting the BM ROI from the total lesion ROI. BM and VE volumes were calculated by summing up the ROIs and then multiplied by the corresponding slice interval. Confluent VE (connecting edemas from more than one BM, n = 320/865) were considered as edema positive but were not included in calculations of VE volumes. Accuracy of lesion masks was evaluated and corrected if applicable by two independent readers (S. S. and T. S. with ten and three years of experience in Neuroradiology, respectively).

### Statistical analysis

Statistical analysis was conducted using R software (R version 3.3.1; The R Foundation for Statistical Computing) and IBM SPSS Statistics^®^ (IBM^®^ 2011, version 22, Armonk, New York, USA). For univariate analysis, a dependent t-test (correlation of BM and non-confluent VE size), Mann-Whitney U test (comparison of sizes of edema-positive and edema-negative BM and volumes of supra- and infratentorial BM), or Pearson’s chi-squared test (occurrence of non-confluent VE supra- and infratentorial) was used. Based on the hypothesis that edema formation is associated with metastases volume, we then performed a logistic regression analysis using a generalized mixed-effects model with BM volume as predictor and edema formation (dichotomous variable edema-positive/ edema-negative) as response variable, including patient identification number as random-effect. Based on this model, receiver-operating characteristic analysis was used to calculate optimal threshold values of BM volume for VE formation including all edema-negative BM (n = 551) and BM with non-confluent edema (n = 545). To minimize the number of false negative cases (large BM without VE) and false positive cases (small BM with VE), the threshold with both maximum sensitivity and specifity (Youden-index) was calculated. To provide clinical applicability, corresponding diameters were calculated for each determined threshold value. Assuming that BM are shaped spherically, we computed the BM diameters by using the sphere formula ($\text{V}=\frac{4}{3}{\pi \text{r}}^{3}$).

Based on the hypothesis that edema formation might not only be dependent on BM volume but also on location (supra-/ infratentorial) and primary tumor we used mixed-effects modeling again with the model mentioned above. We added localization (supra-/infratentorial) and the five largest groups of primary tumors first as single variables and as interaction terms. A P-value < 0.05 was considered significant. If not otherwise indicated, data are given as median and interquartile range in parenthesis.

## Results

### Descriptive statistics

The median age at initial diagnosis of the primary neoplasm was 62 (53–72) years. BM developed with a latency of 9 (0–33.5) months. The primary tumor types and number of metastases were as follows: pulmonary (number of patients: 85/number of BM: 799; tumor subtype: 61 non-small cell lung cancer, 24 small cell lung cancer), genitourinary (GU; 20/68: 8 kidney, 5 prostate, 2 ovarian, and 2 testicular cancer, 1 choriocarcinoma, 1 urothelial cell, and 1 uterine cancer), skin (20/62: 18 melanoma, 1 Merkel-cell, and 1 squamous cell carcinoma), breast (18/269), gastrointestinal (GI; 17/97: 6 esophageal, 4 colon, 4 rectal, 2 sigmoidal, and 1 gastric cancer), cancer of unknown primary (CUP; 6/47), sarcoma (2/20: 1 desmoplastic small round cell tumor and 1 endometrial stromal sarcoma), and head and neck (1/2: 1 thyreoid cancer).

The highest number of BM per patient at first diagnosis was found in breast cancer (3 (2–12) BM per patient) followed by lung cancer (3 (1–6)). Table 1 shows the distribution and size of BM and VE among all patients and by primary neoplasm.

**Table 1:** Distribution and size of BM and VE among the whole cohort and the different primary tumors.

	All	Pulmonary	GU	Skin	Breast	GI	CUP	Sarcoma	Head/neck
**No. of patients**	173	85	20	20	18	17	10	2	1
**No. of BM**	1416	799	68	62	269	97	99	20	2
**No. of VE**^1^	865	397	56	47	199	75	71	19	1
** • Supratentorial**	1016	664	63	56	77	65	69	20	2
** • Infratentorial**	400	135	5	6	192	32	30	0	0
**Median (IQR) no. of BM per patient**	2 (1–6)	3 (1–6)	1 (1–2.75)	2 (1–3)	3 (2–12)	2 (1–6.5)	1 (1–18)	10	1
**No. of patients with solitary BM**	61	25	12	9	3	5	6	1	0
**Median (IQR) BM size in ml**	0.07 (0.02–0.25)	0.07 (0.02–0.23)	0.12 (0.06–0.76)	0.05 (0.01–0.37)	0.04 (0.01–0.13)	0.08 (0.03–0.26)	0.11 (0.04–0.42)	0.48 (0.21–0.98)	0.14
**Median (IQR) VE size**^2^ **in ml**	0.47 (0.14–2.18)	0.51 (0.14–2.48)	2.32 (0.33–11.28)	0.86 (0.15–2.26)	0.27 (0.08–1.02)	1.05 (0.22–18.06)	0.19 (0.09–1.74)	0.31 (0.14–1.15)	0.51

GU, genitourinary primary tumor; CUP, cancer of unknown primary; GI, gastrointestinal primary neoplasm; BM, brain metastases; VE, vasogenic edema; IQR, interquartile range

^1^ Non-confluent and confluent VE.

^2^ Non-confluent VE only.

### Association of BM volume and VE formation

For all BM with non-confluent VE, there was a positive, statistically significant correlation between BM volume and VE volume (*t*(544) = -7.364, P < 0.001). BM with non-confluent edema showed significantly higher volumes compared to BM without edema (U = 69371.5, P < 0.001). When using the mixed effects model, BM volume was also significantly associated with VE formation (P < 0.001). The primary tumor type had no influence on VE formation in the simple model, but the influence become significant in the interaction model.

### Edema formation threshold

**Fig 2:**
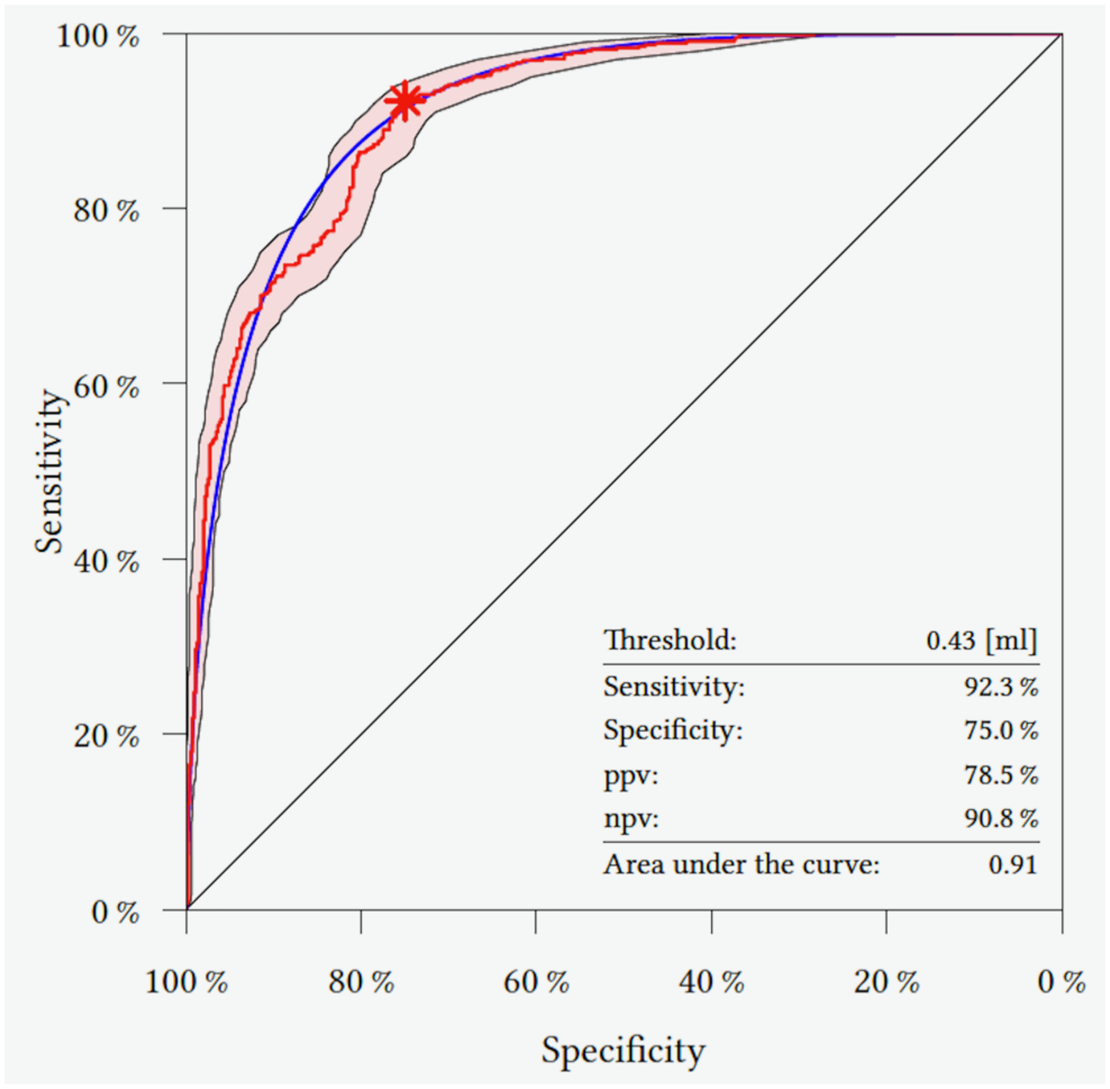
ROC curve determining the best threshold (maximum sensitivity and maximum specifity) for development of vasogenic edema of all BM in the study (red star = 0.48 ml). The red line represents the empirical ROC curve, the light red area shows the 95% confidence interval, and the blue line is a smoother of the empiric ROC curve. Sensitivity, specifity, positive predictive value (ppv), negative predictive value (npv), and area under the curve are also given.

Table 2 shows the optimal cut-off for BM diameter above which edema occurred. It was 9.37 mm for all, 9.06 mm for pulmonary, 26.98 mm for GU, 36.0 mm for skin, 13.11 mm for breast, 4.57 mm for GI, and 26.98 mm for CUP primary tumors (Fig 2). Corresponding sensitivities, specificities, positive predicitive value, an, and area und the curve are given in Table 2.

**Table 2:** Overview of thresholds for BM formation of all patients and the different primary tumors.

BM	Cut-off (in mm / ml)	Sensitivity (%)	Specificity (%)	PPV (%)	NPV (%)	AUC
**All**	9.37 / 0.43	92.3	75.0	78.5	90.8	0.91
**Pulmonary**	9.06 / 0.39	90.2	82.3	78.4	92.2	0.93
**GU**	26.98 / 10.28	93.2	75.0	93.2	75.0	0.86
**Skin**	36.00/ 24.43	80.0	100.0	100.0	71.4	0.96
**Breast**	13.11 / 1.18	90.3	87.1	87.8	89.7	0.92
**GI**	4.57 / 0.05	80.4	59.1	80.4	59.1	0.74
**CUP**	8.02 / 0.27	97.9	85.7	92.0	96.0	0.94

AUC, area under the curve; BM, brain metastases; CI, confidence interval; GU, genitourinary primary tumor; GI, gastrointestinal primary tumor; PPV, positive predictive value; NPV, negative predictive value

### Dependence on localization

BM tend to reside infratentorially, whereas lung, GU, and skin metastases favor the supratentorial space (Table 1). Supratentorial BM did not show higher volumes than infratentorial BM (U = 100455.0, P = 0.194). In the infratentorial space, metastases that exhibited edema were more frequent than those without edema, as opposed to the supratentorial space (BM without VE > BM with VE), χ(1) = 9.375, P = 0.002. When using the mixed effects model, BM location neither had an influence on VE formation in the simple nor in the interaction model and therefore, no threshold for VE development was calculated.

## Discussion

To the best of our knowledge, this is the first radiological study evaluating the development of VE in patients with BM. We were able to demonstrate that VE development is dependent on the volume of the underlying BM and the site of the primary neoplasm, but not from the location of the BM. The “best” threshold for edema development was 9.4 mm for all patients and differed for the distinct primary tumor groups.

The development of BM is most commonly due to hematogeneous dissemination of cancer cells with subsequent growth in the brain parenchyma. The biology of cancer cell migration to the brain is poorly understood. Proposed factors influencing the site of metastases formation are summarized under the “seed vs. soil hypothesis”, which incorporates the biochemical environment including cerebral cell surface properties and the “anatomical-mechanical hypothesis”, which describes the influence of local blood flow attributes and vessel size [11, 12]. As an example, we found that the majority of posterior fossa metastases were of breast or pulmonary origin whereas skin cancer BM favored the supratentorial space, which is in line with previous studies [13–15].

Numerous factors are involved in the pathophysiology of VE formation surrounding BM. For example, during the process of tumor-mediated angiogenesis, BM recruit healthy endothelial cells from surrounding brain parenchyma, but the vascularization process is disturbed by tumor-induced lack of transmembrane tight junction proteins occludin, claudin-1, and claudin-5 [16, 17]. As a consequence, interendothelial tight junctions become defective, causing increased permeability of the blood-brain barrier [3]. In contrast, reactive astrogliosis surrounding the metastasis was shown to decrease fluid penetration restricting the edema [18]. VE elimination seems to be dependent on aquaporin-4, a water channel mainly expressed in the brain [3]. The extent of tumor-infiltrating lymphocytes also influences VE formation. Berghoff et al. demonstrated that greater CD3+/CD8+-T cell infiltration correlated with peritumoral edema formation and was also associated with a prolonged median overall survival [19]. This observation highlights the potential role of local cytokine production and the tumor-specific immune response for edema formation. Taken together, the fluid inflow-outflow balance of the extracellular brain parenchyma is shifted towards influx in VE, resulting in increased intracranial pressure [3, 20, 21]. As a general rule, VE preferably occurs in the white matter, which contain radially oriented nerve fibers rather than densely packed nerve cell bodies of the grey matter.

Sekine et al. demonstrated differences in BM count, BM sizes, and VE volumes in 57 non-small cell lung cancer patients with differences in *EGFR* mutation status [22]. Hengel et al. reported that BM from lung cancer are more likely to show an edema at >3–4 cm in diameter compared to BM from breast cancer, but the size above which a BM initiates VE formation has not been further studied [14]. Since we demonstrated that the general minimum cut-off value for the presence of VE is a BM diameter of 9.4 mm, but differs between primary sites, we hypothesize that the above-mentioned mechanisms leading to an edema are histology-specific. Further pathologic and molecular studies are needed to understand the different processes involved in edema formation around BM.

Our findings may have some important clinical implications: the absence of FLAIR/T2w hyperintensities indicating edema does not rule out BM, since we found altogether 3/1096 false negative BM. However, the volume of VE provides information about the size of the underlying metastasis. This may become relevant in already diagnosed BM patients, in whom administration of contrast agent must be avoided (e. g. due to kidney failure), but who may need to undergo MRI to evaluate therapy effects. Additionally, our findings may suggest the primary site in patients in whom diagnosis of BM precedes the discovery of the primary tumor.

Our study is mainly limited due to selection and sampling bias, as pathology reports of BM for precise identification of primary site were only available in patients who underwent resection or biopsy of BM. Surgery was performed in a minority of cases with symptomatic space-occupying BM or BM with unknown primary tumor only. However, the histology report of the primary neoplasm was available in all other patients and cases with a history of more than one tumor within the last ten years were excluded. In addition, MRI protocols were not uniform and imaging was performed on different scanner types due to the retrospective character of this study.

## Conclusion

Taken together, we found that VE development mainly depends on the volume of the underlying BM and the site of the primary neoplasm, but not from the location of the BM. On initial diagnosis, administration of contrast agent on MRI should be mandatory for diagnoses of metastases less than 9–10 millimeters in diameter. Edema volume indicates size of underlying BM, which may be helpful in follow-up imaging without contrast. Knowledge of VE and BM volume could help predict the primary tumor type in patients where discovery of the BM precedes that of the primary site. The differential impact of tumor histology on edema extent is a fertile topic for future investigations on the pathophysiology of tumor mediated edema formation.

## Supporting information

S1 Table Patient characteristics. (SAV)

## References

[1] Walker AE, Robins M, Weinfeld FD. Epidemiology of brain tumors: the national survey of intracranial neoplasms. Neurology. 1985;35(2):219–26. 3969210 10.1212/wnl.35.2.219

[2] Criscuolo GR. The genesis of peritumoral vasogenic brain edema and tumor cysts: a hypothetical role for tumor-derived vascular permeability factor. Yale J Biol Med. 1993;66(4):277–314. 7516104 PMC2588896

[3] Papadopoulos MC, Saadoun S, Binder DK, Manley GT, Krishna S, Verkman AS. Molecular mechanisms of brain tumor edema. Neuroscience. 2004;129(4):1011–20. 10.1016/j.neuroscience.2004.05.044 15561416

[4] Drappatz J. Management of vasogenic edema in patients with primary and metastatic brain tumors. In: UpToDate, Post TW (Ed), UpToDate, Waltham, MA. (Accessed on August 10, 2016.).

[5] Schaefer PW, Budzik RF Jr., Gonzalez RG. Imaging of cerebral metastases. Neurosurg Clin N Am. 1996;7(3):393–423. 8823771

[6] Runge VM. Contrast-Enhanced Clinical Magnetic Resonance Imaging. Lexington: The University Press of Kentucky; 1997.

[7] Bravo-Rodríguez F, Diaz-Aguilera R, Hygino da Cruz LC Jr.. Learning Neuroimaging: 100 Essential Cases (Learning Imaging). Berlin Heidelberg: Springer-Verlag; 2012.

[8] Aminoff A, Josephson A. Aminoff's Neurology and General Medicine. Oxford: Elsevier; 2014.

[9] Ramalho J, Semelka RC, Ramalho M, Nunes RH, AlObaidy M, Castillo M. Gadolinium-Based Contrast Agent Accumulation and Toxicity: An Update. AJNR Am J Neuroradiol. 2016;37(7):1192–8. 10.3174/ajnr.A4615 26659341 PMC7960350

[10] Robb RA. The biomedical imaging resource at Mayo Clinic. IEEE Trans Med Imaging. 2001;20(9):854–67. 10.1109/42.952724 11585203

[11] Paget S. The distribution of secondary growths in cancer of the breast. 1889. Cancer Metastasis Rev. 1989;8(2):98–101. 2673568

[12] Ewing J. Neoplastic Diseases: A Treatise on Tumors. 3rd ed Philadelphia: WB Saunders; 1928.

[13] Quattrocchi CC, Errante Y, Gaudino C, Mallio CA, Giona A, Santini D, et al Spatial brain distribution of intra-axial metastatic lesions in breast and lung cancer patients. J Neurooncol. 2012;110(1):79–87. 10.1007/s11060-012-0937-x 22802020

[14] Hengel K, Sidhu G, Choi J, Weedon J, Nwokedi E, Axiotis CA, et al Attributes of brain metastases from breast and lung cancer. Int J Clin Oncol. 2013;18(3):396–401. 10.1007/s10147-012-0392-x 22383025

[15] Rogne SG, Helseth E, Brandal P, Scheie D, Meling TR. Are melanomas averse to cerebellum? Cerebellar metastases in a surgical series. Acta Neurol Scand. 2014;130(1):1–10. 10.1111/ane.12206 24313862

[16] Wolburg H, Lippoldt A. Tight junctions of the blood-brain barrier: development, composition and regulation. Vascul Pharmacol. 2002;38(6):323–37. 12529927 10.1016/s1537-1891(02)00200-8

[17] Papadopoulos MC, Saadoun S, Woodrow CJ, Davies DC, Costa-Martins P, Moss RF, et al Occludin expression in microvessels of neoplastic and non- neoplastic human brain. Neuropathol Appl Neurobiol. 2001;27(5):384–95. 11679090 10.1046/j.0305-1846.2001.00341.x

[18] Roitbak T, Sykova E. Diffusion barriers evoked in the rat cortex by reactive astrogliosis. Glia. 1999;28(1):40–8. 10498821 10.1002/(sici)1098-1136(199910)28:1<40::aid-glia5>3.0.co;2-6

[19] Berghoff AS, Fuchs E, Ricken G, Mlecnik B, Bindea G, Spanberger T, et al Density of tumor-infiltrating lymphocytes correlates with extent of brain edema and overall survival time in patients with brain metastases. Oncoimmunology. 2016;5(1):e1057388 10.1080/2162402X.2015.1057388 26942067 PMC4760339

[20] Monro A. Observations on the structure and function of the nervous system. Edinburgh: Creech & Johnson; 1823.

[21] Kellie G. An account of the appearances observed in the dissection of two of the three individuals presumed to have perished in the storm of the 3rd, and whose bodie were discovered in the vicinity of Leith on the morning of the 4th November 1821 with some reflections on the pathology of the brain In: The Transactions of the Medico-Chirurgical Society of Edinburgh. pp. 84–169. PMC5405298 29583621

[22] Sekine A, Kato T, Hagiwara E, Shinohara T, Komagata T, Iwasawa T, et al Metastatic brain tumors from non-small cell lung cancer with EGFR mutations: distinguishing influence of exon 19 deletion on radiographic features. Lung Cancer. 2012;77(1):64–9. 10.1016/j.lungcan.2011.12.017 22335887

